# New Insight into the Reactivity of S,S-Bis-ylide

**DOI:** 10.3390/molecules28083295

**Published:** 2023-04-07

**Authors:** Ugo Authesserre, V. S. V. S. N. Swamy, Nathalie Saffon-Merceron, Antoine Baceiredo, Tsuyoshi Kato, Eddy Maerten

**Affiliations:** 1 Université de Toulouse, UPS, and CNRS, LHFA UMR 5069, 118 Route de Narbonne, 31062 Toulouse, France; 2 Université de Toulouse, UPS, and CNRS, ICT UAR2599 118 Route de Narbonne, 31062 Toulouse, France

**Keywords:** bis-ylides, ylide, carbone, phosphenium, carbene, carbon dioxide

## Abstract

The present work focuses on the reactivity of S,S-bis-ylide **2**, which presents a strong nucleophilic character, as evidenced by reactions with methyl iodide and CO_2_, affording C-methylated salts **3** and betaine **4**, respectively. The derivatization of betaine **4** affords the corresponding ester derivative **6**, which is fully characterized by using NMR spectroscopy and X-ray diffraction analysis. Furthermore, an original reaction with phosphenium ions leads to the formation of a transient push–pull phosphino(sulfonio)carbene **8**, which rearranges to give stabilized sulfonium ylide derivative **7**.

## 1. Introduction

**Figure 1:**
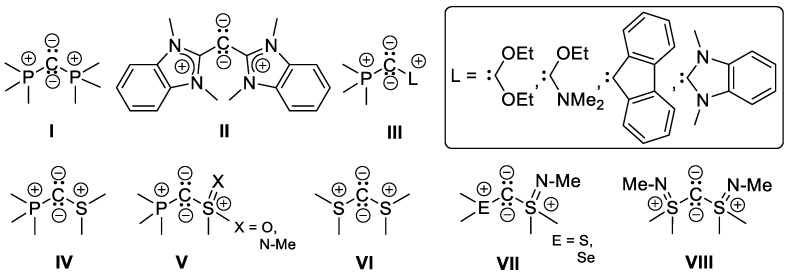
Carbodiphosphoranes **I**, carbodicarbenes **II**, carbophosphinocarbenes **III**, phosphonio/sulfonio bis-ylides **IV**, phosphonio/sulfoxonio or phosphonio/iminosulfonio bis-ylides **V**, sulfonio/sulfonio bis-ylides **VI**, selenio- or sulfonio-/iminosulfonio **VII,** and bis iminosulfanebis-ylides **VIII**.

Since the discovery of ylides in the 1920s [1,2], their chemistry has experienced important growth [3,4,5], and they have become very important synthetic tools [6,7,8,9]. Bis-ylides, which are the combination of two ylide functions on a single carbon atom, have attracted significant attention from many research groups [10,11,12,13,14,15,16]. Indeed, since the first synthesis of carbodiphosphoranes **I** by Ramirez in the 1960s [17], many models have been developed (Figure 1). Among them, we can mention carbodicarbenes **II** prepared by Bertrand’s group [18], after initial theoretical predictions by Frenking [19]; mixed carbophosphinocarbene **III**, initially synthesized by Fürstner and Alcarazo and later by Ong et al. [20,21,22]; and mixed phosphonio/sulfonio **IV** and phosphonio/sulfoxonio **V** (X = O) bis-ylides prepared by our group [23,24,25]. Finally, major contributions were made by Fujii’s group with various combinations of selenium/sulfur- or sulfur/sulfur-stabilized carbones **V**–**VIII** [26,27,28,29,30]. Naturally, the diversity of these structures has brought considerable knowledge on these species, which exhibit very diverse reactivity, ranging from their use as ligands in organometallic chemistry [15] to the activation of small molecules [21] and even their application as atomic carbon sources [23]. The main application of bis-ylides remains their use as ligands because of the peculiar environment of the central carbon atom. In particular, the presence of two lone pairs located on a single carbon atom [10,11,12,13,14,15,16] offers unique coordination modes for transition metals. Indeed, Stephen took advantage of the presence of the two lone pairs to stabilize certain transition metals, achieving high efficiency in hydrogenation reactions [31]. Ong’s group has evidenced that the unexpected accepting character of carbodicarbenes at the N-heterocyclic carbene fragments was beneficial for ambiphilic-type reactivity allowing the activation of small molecules [32,33]. It is obvious that the reactivity and applications of bis-ylides are directly linked to the combination of the two ylide functions, but overall remain rare.

In 2010, we reported the first stable and isolable mixed P,S-bis-ylide **IV** [23], which can be used as an atomic carbon reagent to introduce one carbon atom into various organic molecules [34]. In particular, the labile character of the sulfide ligand in **IV** facilitates substitution reactions at the central carbon atom and, thus, atomic carbon transfer reactions. Therefore, S,S-bis-ylide **VI** consisting of two labile sulfide moieties should be an excellent atomic carbon reagent. However, probably due to the thermal instability of **VI**, only a few reactions with Au(I), demonstrating its carbone character, were described [30], and no reaction with organic reagents has been reported to date. In this article, we report the original reactions of S,S-bis-ylide **VI** with CO_2_ and phosphenium ions (Figure 1).

## 2. Results and Discussion

### 2.1. Synthesis

**Scheme 1:**
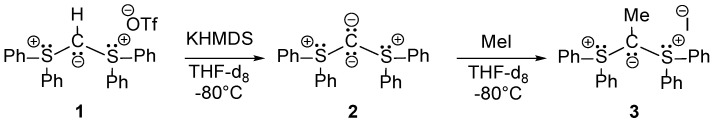
Synthesis of S,S-bis-ylide **2** and alkylation reaction with MeI.

Monoprotonated precursor **1** (Scheme 1) was prepared according to the method previously described by Fujii [30]. The spectroscopic data are in good agreement with the reported values. In particular, the central proton and carbon resonances of **1** appear at δ = 3.88 ppm and δ = 19.4 ppm, respectively, in the ^1^H and ^13^C NMR spectra. Because of the thermal instability of S,S-bis-ylide **2**, the deprotonation was then realized in THF-d_8_ using potassium hexamethyldisilazane at −80 °C and was analyzed at low temperature, in situ, in the presence of conjugated acid. With the appearance of a bright-yellow color, in the ^1^H NMR spectrum, the C-H signal disappeared, while the central carbon in the ^13^C NMR spectrum was lower-field-shifted at δ = 39.0 ppm compared to **1**. Bis-ylide **2** starts to decompose at −30 °C to afford black carbon precipitate and diphenyl sulfide.

### 2.2. Reactivity

**Figure 2:**
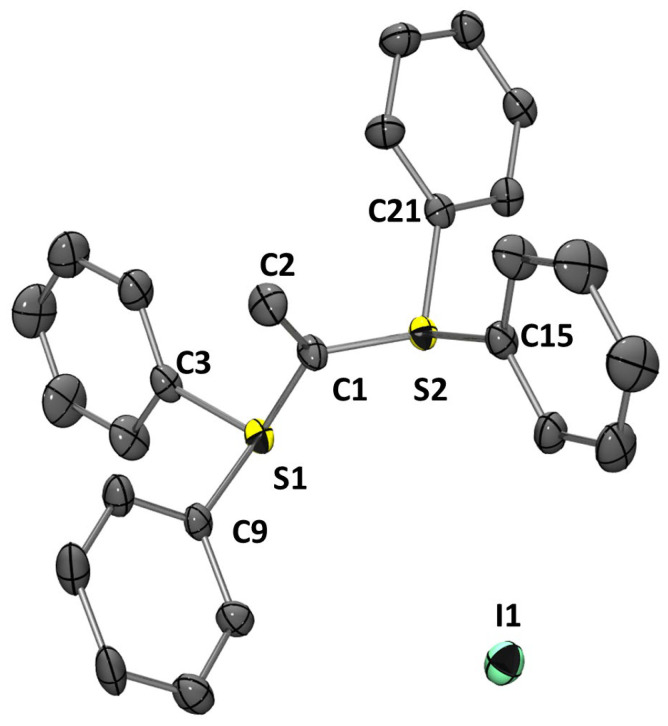
Molecular structure of C-methylated salt **3**. Ellipsoids are drawn at the 30% probability level; hydrogen and disordered atoms are omitted for clarity. Selected bond lengths (Å) and angles (°): S1–C1 1.682(9), C1–S2 1.687(9), C1–C2 1.525(12), S1–C3 1.794(4), S1–C9 1.759(3), S2–C21 1.772(4), S2–C15 1.776(3). S1–C1–S2 111.6(6), S2–C1–C2 123.8(6), C2–C1–S1 124.3(6), C9–S1–C1 107.4(3), C1–S1–C3 111.6(4), C3–S1–C9 100.2(2), C1–S2–C15 107.6(3), C15–S2–C21 101.5(2), C21–S2–C1 111.9(4).

The formation of S,S-bis-ylide **2** was unambiguously confirmed by its methylation reaction (Scheme 1). Indeed, upon the addition of one equivalent of methyl iodide to a THF solution of **2** at −80 °C, the yellow color immediately disappeared concomitantly with the precipitation of a white solid. After treatment, the desired C-methylated salt **3** was isolated in an 85% yield. The methylation was confirmed by the presence of two characteristic signals for the methyl group at δ = 1.67 ppm and δ = 10.2 ppm in the ^1^H and ^13^C NMR spectra, respectively (see Supplementary Materials). C-methylated salt **3** was isolated in crystalline form by layering diethyl ether onto a saturated dichloromethane solution, and its structure was confirmed by X-ray diffraction analysis (Figure 2). As expected, the S-C bond lengths in **3** (1.682 and 1.687 Å) are very similar to those of the protonated bis-ylide precursor **1** (1.685 Å and 1.676 Å) [30] and much shorter than an S-C single bond length (1.81 Å), showing the delocalization of the negative charge from the central carbon atom to the SPh_2_ fragments and justifying the planar environment around the C1 atom (∑°= 359.7°). The S1-C1-S2 angle is also almost identical to that of **1**.

**Scheme 2:**
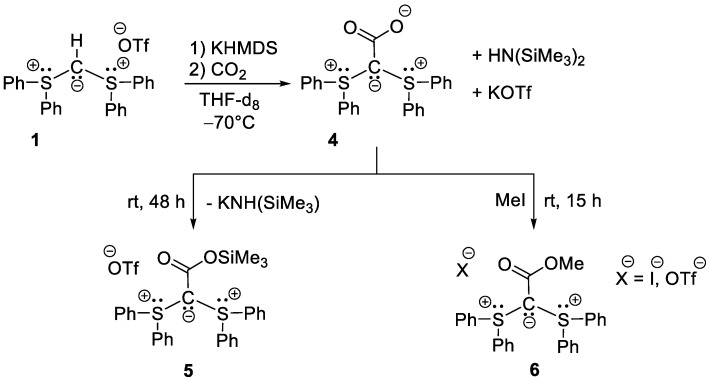
Reactivity of S,S-bis-ylide **2** with CO_2_ and corresponding esters **5** and **6**.

**Figure 3:**
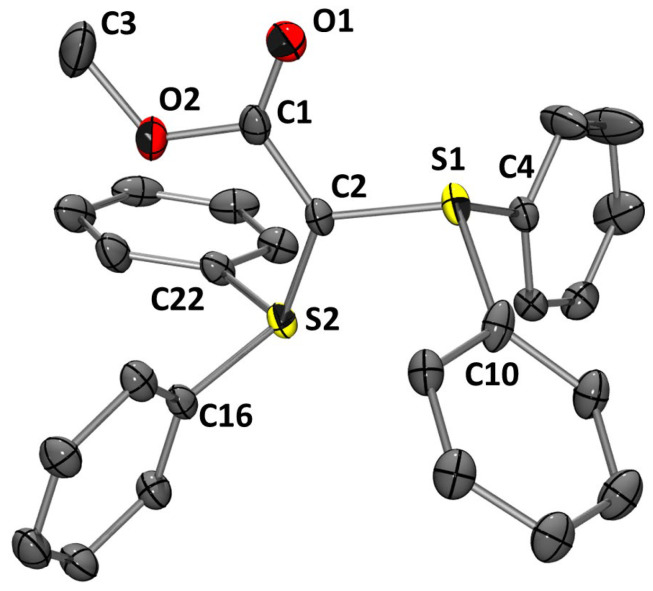
Cationic part of **6**. Ellipsoids are drawn at the 30% probability level; hydrogen and disordered atoms and counter ion (I^−^) are omitted for clarity. Selected bond lengths (Å) and angles (°): S1–C2 1.720(2), C2–S2 1.713(3), C1–C2 1.437(4), C1–O1 1.214(3), C1–O2 1.351(3), O2–C3 1.438(4), S1–C4 1.783(3), S1–C10 1.803(5), S2–C22 1.793(3), S2–C16 1.794(3). S1–C2–S2 117.5(1), S2–C2–C1 127.6(2), C1–C2–S1 114.1(2), C2–C1–O2 110.8(2), O2–C1–O1 122.6(3), O1–C1–C2 126.6(2), C2–S1–C4 109.0(2), C2–S1–C10 106.6(6), C4–S1–C10 104.7(7), C2–S2–C22 108.5(2), C2–S2–C16 110.2 (2), C22–S2–C16 103.8(1).

It is well known that carbodiphosphoranes (P,P-bis-ylides) react with CO_2_ to form zwitterionic donor–acceptor complexes that can coordinate transition metals or evolve upon heating toward phosphine oxide and phosphoranylideneketene [35,36,37,38,39]. The sulfur analog **2** also reacts with CO_2_ (3 bars) at −70 °C, affording the corresponding CO_2_ adduct **4** (Scheme 2). ^13^C NMR analysis of the resulting solution at −20 °C shows two broad signals appearing at δ = 167.2 ppm and δ = 40.16 ppm, corresponding to the coordinated CO_2_ fragment and the central carbon atom, respectively, which is in good agreement with the formation of betaine **4**. However, at room temperature, zwitterionic CO_2_ adduct **4** reacts slowly with the hexamethyldisilazane byproduct to afford the O-silylated ester **5** [40]. Alternatively, CO_2_ adduct **4** can be trapped by adding one equivalent of MeI to generate the O-methylated salt **6**, which is stable and was easily isolated in an 80% yield. Compound **6** was fully characterized by NMR spectroscopy (see experimental section and supporting information). The single crystals of **6** were grown by layering pentane onto a saturated DCM at room temperature [41] and then analyzed by X-ray diffraction, confirming the expected structure (Figure 3). The S1–C2 and S2–C2 bond lengths (1.720(2) Å and 1.713(3) Å, respectively) are slightly longer than those of **3** (1.682 Å and 1.687 Å). The central carbon C1 shows a planar environment, and the C1–C2 bond length is short 1.437(4) Å. This bonding pattern can be explained by a major contribution of the ester fragment to stabilize the negative charge [42].

**Scheme 3:**
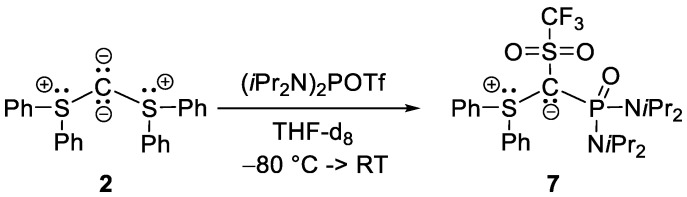
Reactivity of S,S-bis-ylide **2** with a phosphenium ion.

**Figure 4:**
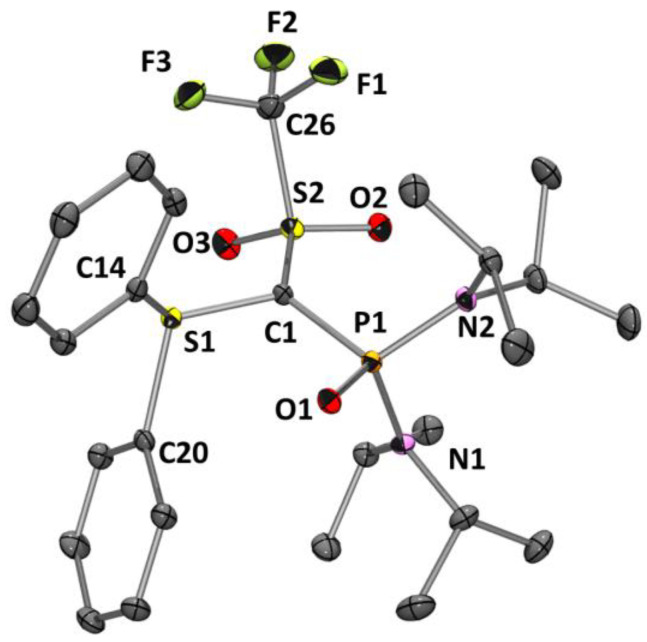
Molecular structure of **7**. Ellipsoids are drawn at the 30% probability level; hydrogen atoms are omitted for clarity. Selected bond lengths (Å) and angles (°): S1–C1 1.724(2), C1–S2 1.694(2), C1–P1 1.807(2), S1–C14 1.803(2), S1–C20 1.788(2), P1–O1 1.487(1), P1–N1 1.669(2), P1–N2 1.656(2), S2–O2 1.434(2), S2–O3 1.437(1), S2–C26 1.841(2), C26–F1 1.327(2), C26–F2 1.336(2), C26–F3 1.333(2). S1–C1–P1 120.80(10), S1–C1–S2 109.97(10), P1–C1–S2 127.57(11), C1–S1–C20 112.86(9), C1–S1–C14 110.71(9), C20–S1–C14 105.04(9).

**Scheme 4:**
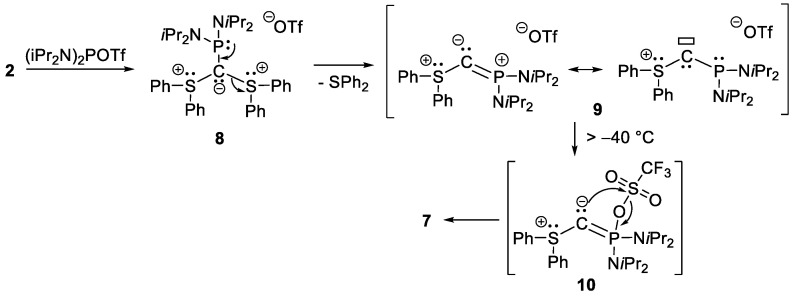
Proposed mechanism.

In order to demonstrate the leaving group ability of the sulfide group, we considered the reaction of S,S-bis-ylide **2** with an electrophile bearing a lone pair such as a phosphenium ion (Scheme 3) [43]. The reaction of **2** with bis(diisopropylamino)phosphenium ion at −80 °C affords ylide **7** with good selectivity when the reaction mixture is rapidly brought to room temperature [44]. The structure of **7** was established by using X-ray diffraction analysis (Figure 4). Variable temperature NMR analysis indicated the formation of adduct **8** (δ = 48.3 ppm in ^31^P NMR) at −70 °C, which starts to evolve towards sulfonium ylide **7** (δ = 25.7 ppm in ^31^P NMR) above −40 °C (carbene intermediate **9** was not detected). The process can be explained by the initial attack of nucleophilic bis-ylide **2** on the positively charged phosphenium salt generating a phosphino cationic ylide intermediate **8** (Scheme 4). The π-donation of the phosphine lone pair induces sulfide elimination and the formation of phosphino(sulfonio)carbene **9**. This push–pull carbene, substituted by a π-donating phosphino- and a π-accepting sulfonio-group, exhibits a strong electrophilic character on the phosphorus center [45] and, therefore, reacts with triflate anion to afford **10**. Finally, rearrangement, starting with the nucleophilic attack of central carbon to the sulfoxide moiety, occurs to afford sulfonium ylide **7,** which is stabilized by two π-accepting substituents, such as trifluoromethanesulfonyl and phosphonyl groups, as indicated by the short C1–S2 and C1–P1 bond lengths (1.694(2) and 1.807(2) Å, respectively). Thanks to the high leaving group ability of sulfide ligand, such a strongly electrophilic phosphino(sulfonio)carbene **9**, which reacts even with the generally non-coordinating triflate anion, can be generated from **8** by the elimination of diphenyl sulfide. It should be noted that, in the case of carbodiphosphoranes, such an addition–elimination reaction does not take place. Instead, they react with chlorophosphine to form a stable adduct similar to **8** [46,47].

## 3. Materials and Methods

### 3.1. General Comments

All manipulations were performed under an inert atmosphere of argon by using standard Schlenk techniques or high-pressure NMR tube techniques. Solvents were purified with an MBraun SBS-800 purification system. Dry and oxygen-free solvents were used. ^1^H, ^13^C, ^19^F, and ^31^P NMR spectra were recorded on Brucker Avance II 300 MHz, Avance III HD 400 MHz, and Avance I and II 500 MHz spectrometers (Brucker, Karlsruhe, Germany). Chemical shifts were expressed in parts per million with residual solvent signals as internal reference (^1^H and ^13^C{^1^H}). ^19^F and ^31^P NMR chemical shifts were reported in ppm relative to CFCl_3_ and 85% H_3_PO_4_, respectively. The following abbreviations and their combinations were used: br—broad; s—singlet; d—doublet; t—triplet; q—quartet; hept—heptuplet; m—multiplet. ^1^H and ^13^C resonance signals were attributed by means of 2D COSY, HSQC, and HMBC experiments. Mass spectra were recorded on a Hewlett Packard 5989A spectrometer (Hewlett-Packard, Palo Alto, CA, USA). High-resolution MS (HRMS) spectra were realized on a Xevo G2 QTof apparatus (Waters, Milford, CT, USA). Melting points were measured in a sealed glass tube on a Stuart SMP-30 automatic melting point apparatus. All commercially available reagents were used without further purification otherwise noted. Moreover, **1** was prepared following a previously reported procedure [30].

### 3.2. Synthesis

Protonated precursor of bis-ylide **1**: The product was synthesized following previously reported procedure [30]. ^1^H NMR (THF-_d8_, 298 K, 500 MHz): δ = 4.91 (s, 1H, SC*H*S), 7.41–7.49 (m, 12H, C*H*_ar_), 7.89–7.94 (m, 8H, C*H*_ar_) ppm; ^1^H NMR (CD_3_CN, 298 K, 300 MHz): δ = 3.90 (s, 1H, SC*H*S), 7.53–7.67 (m, 20H, C*H*_ar_) ppm; ^13^C{^1^H} NMR (CD_3_CN, 298 K, 126 MHz): δ = 19.4 (s, S*C*HS), 122.2 (q, *J*_CF_ = 321 Hz, *C*F_3_), 128.7 (s, *C*H_ar_), 131.4 (s, *C*H_ar_), 133.2 (s, *C*H_ar_), 137.0 (s, *C_ipso_*) ppm; ^13^C{^1^H} NMR (THF-*d*_8_, 298 K, 126 MHz): δ = 15.8 (s, S*C*HS), 122.2 (q, *J*_CF_ = 322.1 Hz, *C*F_3_), 128.4 (s, *C*H_ar_), 130.9 (s, *C*H_ar_), 132.3 (s, *C*H*_para_)*, 138.2 (s, *C_ipso_*) ppm; ^19^F{^1^H} NMR (THF-*d*_8_, 298 K, 282 MHz): δ = −78.7 (s) ppm. Mp = 144 °C. HRMS (ESI+): *m*/*z* [M]^+^ calculated for C_25_H_21_S_2_ = 385.1085 found = 385.1082.

S,S-Bis-ylide **2**: In a J. Young NMR tube, the protonated precursor of bis-ylide **1** (50 mg, 0.094 mmol, 1 eq.) was dissolved in THF-*d*_8_ (0.2 mL). At –80 °C, a solution of KHMDS (19 mg, 0.095 mmol, 1 eq.) in 0.2 mL of THF-*d*_8_ was added. The NMR tube was then closed, and the mixture was carefully shaken while remaining at a low temperature. A yellow-colored solution was obtained. Because of the thermal instability of **2**, the product was used or characterized at a low temperature in the presence of hexamethyldisilazane without any purification. ^1^H NMR (400 MHz, 213 K, THF-*d*_8_): δ = 7.41–7.26 (m, 12H, C*H*_ar_), 8.02–7.90 (m, 8H, C*H*_ar_) ppm. ^13^C{^1^H} NMR (101 MHz, 213 K, THF-*d*_8_): δ = 39.0 (s, S*C*S), 127.1 (s, *C*H_ar_), 129.3 (s, *C*H_ar_), 129.6 (s, *C*H_para_), 148.2 (s, *C*_ipso_) ppm.

C-methylated salt **3**: In a Schlenk flask containing bis-ylide precursor **1** (50 mg, 0.094 mmol, 1 eq.) and KHMDS (19 mg, 0.095 mmol, 1 eq.), 0.5 mL of THF was added at −80 °C. After the yellow coloration of the solution, 1 equivalent of iodomethane (5.9 µL, 0.094 mmol, 1 eq.) was carefully added at the same temperature. The solution immediately became colorless, and a white precipitate appeared. After 15 min, the solution was allowed to warm up to RT, then the volatiles were carefully removed under reduced pressure. The crude was extracted with CH_2_Cl_2_ (3 × 2 mL). The resulting solution was dried using MgSO_4_, then evaporated to afford **3** as a white solid (42 mg, 0.080 mmol, 85% yield). Crystals were grown by layering diethyl ether onto a saturated CH_2_Cl_2_ solution. ^1^H NMR (500 MHz, 298 K, CD_3_CN): δ = 1.67 (s, 3H, CH_3_), 7.54–7.57 (m, 8H, C*H*_ar_), 7.59–7.69 (m, 12H, C*H*_ar_) ppm. ^13^C{^1^H} NMR (125 MHz, 298 K, CD_3_CN): δ = 10.2 (s, *C*H_3_), 23.4 (s, S*C*S), 129.7 (s, *C*H_ar_), 131.4 (s, *C*H _ar_), 132.7 (s, *C*_ipso_), 133.3 (s, *C*H_para_) ppm. Mp = 116 °C (decomposition). HRMS (ESI+): *m*/*z* [M]^+^ calculated for C_26_H_23_S_2_ = 399.1241 found = 399.1248.

CO_2_-adduct **4**: In a high-pressure NMR tube, 3 bars of CO_2_ were applied to a solution of bis-ylide **2**, freshly prepared from the protonated precursor **1** (50 mg, 0.094 mmol, 1 eq.) and KHMDS (19 mg, 0.094 mmol, 1 eq.) in THF-*d*_8_ (0.4 mL) at −70 °C. The tube was shaken carefully leading to a colorless solution. Because of the thermal instability of **4**, the product was characterized at 0 °C in the presence of hexamethyldisilazane and CO_2_ without any purification. ^1^H NMR (400 MHz, 273 K, CD_3_CN): δ = 7.30–7.42 (br s, 12H, C*H*_ar_), 7.65–7.88 (br s, 8H, C*H*_ar_) ppm. ^13^C{^1^H} NMR (100 MHz, 273 K, CD_3_CN): δ = 40.2 (br s, S*C*S), 130.7 (s, *C*H_ar_), 130.8 (s, *C*H_ar_), 132.6 (s, *C*H_para_), 134.4 (s, *C*_ipso_), 167.2 (br s, C-*C*O_2_) ppm. 

O-methylated CO_2_ adduct **6**: In a pressure NMR tube, a solution containing freshly generated CO_2_ adduct **4** by using the above-mentioned method at −70 °C then warmed up to room temperature, and CO_2_ pressure was carefully released. Subsequently, MeI (1.0 eq., 6 µL) was added, then precipitation occurred overnight. After the removal of volatiles under reduced pressure, the crude was washed with pentane (3 × 2 mL). Then, **6** was extracted from the crude using DCM. After concentration, the solution was layered with pentane, affording **6** as colorless crystals at room temperature (42 mg, 0.075 mmol, 80% yield). ^1^H NMR (300 MHz, 298 K, CD_2_Cl_2_): δ = 3.67 (s, 3H, C*H*_3_), 7.55–7.65 (m, 16H, C*H*_ar_), 7.66–7.75 (m, 4H, C*H*_ar_) ppm. ^13^C{^1^H} NMR (75 MHz, 298 K, CD_2_Cl_2_): δ = 44.0 (s, S*C*S), 52.6 (s, *C*H_3_), 129.2 (s, *C*_ipso_), 129.7 (s, *C*H_ar_), 131.1 (s, *C*H_ar_), 133.7 (s, *C*H_para_), 164.2 (s, *CO*_2_CH_3_) ppm. Mp = 160 °C (decomposition). HRMS (ESI+): *m*/*z* [M]^+^ calculated for C_27_H_23_O_2_S_2_ = 443.1139 found = 443.1140.

Bis(diisopropylamino)chlorophosphine: 30 mL of PCl_3_ (0.345 mol, 1 eq.) and 50 mL of toluene were introduced into a 1 L bicol fitted with a condenser and an addition funnel. The set-up was cooled with an ice-bath. A mixture of 200 mL of toluene and 275 mL of diisopropylamine (2 mol, 5.7 eq.) was slowly added over 1.5 h, taking particular care of the exothermic character of the reaction. The solution was stirred for an additional 1.5 h at 0 °C, then an additional 15 min at room temperature. A white solid and a yellow solution appeared. The mixture was then heated at reflux for 24 h. Under an argon atmosphere, the resulting solid was placed on a sinter. The ammonium salts were carefully washed with 400 mL of pentane. The resulting red–orange solution was placed under vacuum, affording a red–orange solid. This solid residue was placed, once again, on a sinter and washed with 120 mL of acetonitrile that was previously dried on CaH_2_. The white solid obtained corresponds to the pure desired bis(diisopropylamino)chlorophosphine (76.3 g, 0.286 mol, 83% yield). If some ammonium salts remain in the final product, solubilization of the bis(diisopropylamino)chlorophosphine in pentane followed by filtration of the undesired salts should be performed. ^1^H NMR (300 MHz, 298 K, CDCl_3_): δ = 1.16 (dd, 24H, *J*_HP_ = 21.2 Hz, *J*_HH_ = 6.6 Hz, C*H*_3iPr_), 3.61 (dhept, 4H, *J*_HP_ = 12.7 Hz, *J*_HH_ = 6.6 Hz, C*H(*CH_3_)_2_) ppm. ^31^P{^1^H} NMR (121.5 MHz, 298 K, CDCl_3_): δ = 140.3 ppm. 

Triflate bis(diisopropylamino)phosphenium: In a Schlenk tube, to a solution containing 5.1 g (0.02 mol, 1 eq.) of bis(diisopropylamino)chlorophosphine in 40 mL of dichloromethane at −80 °C was added 3.7 mL (0.02 mol, 1 eq.) of trimethylsilyltrifluromethane sulfonate. After 1 h at –80 °C, the solution was warmed up to room temperature. The volatiles were carefully removed under reduced pressure. The residue was washed twice with diethyl ether (2 × 5 mL). The product obtained was a yellow powder in a 95% yield (6.8 g, 0.019 mol). ^1^H NMR (CDCl_3_, 298 K, 300 MHz): δ = 1.50 (d, ^3^*J*_HH_ = 6.8 Hz, 24H, C*H*_3*i*Pr_), 4.15 (dhept, ^3^*J*_HP_ = 9.9 Hz, ^3^*J*_HH_ = 6.8 Hz, 4H, C*H_i_*_Pr_) ppm; ^13^C{^1^H} NMR (CDCl_3_, 298 K, 75 MHz): δ = 23.8 (d, *J*_PC_ = 8.2 Hz, *C*H_3 *i*Pr_), 52.1 (s, C*H_i_*_Pr_), 119.7 (q, *J*_CF_ = 317.1 Hz, *C*F_3_) ppm; ^31^P{^1^H} NMR (CDCl_3_, 298 K, 122 MHz): δ = 300.5 (s, br) ppm; ^19^F{^1^H} NMR (CDCl_3_, 298 K, 282 MHz): δ = −78.2 (s) ppm.

Sulfonium ylide **7**: In a Schlenk flask containing bis-ylide precursor **1** (50 mg, 0.094 mmol, 1 eq.) and KHMDS (19 mg, 0.094 mmol, 1 eq.) was added 0.5 mL of THF at −80 °C. After the yellow coloration of the solution, 1 equivalent of phosphenium triflate (35.7 mg, 0.094 mmol, 1 eq.) was added at the same temperature. The solution was rapidly brought to room temperature, then the volatiles were removed under reduced pressure. The crude was extracted with CH_2_Cl_2_ (3 × 2 mL). The resulting solution was dried then evaporated. The crude was purified by using flash silica gel chromatography using pentane and ethyl acetate as solvents. Then, **3** was obtained as white solid (32 mg, 0.056 mmol, 60% yield). Crystals can be grown from a saturated CHCl_3_ solution. ^1^H NMR (400 MHz, 298 K, C_6_D_6_): δ = 1.04–1.24 (bs, 12H, C*H*_3_), 1.24–1.34 (d, *J*_PC_ = 7.0 Hz, 12H, C*H*_3_)), 3.80–4.10 (bs, 4H, C*H*), 6.80–7.00 (bs, 6H, C*H*_ar_), 7.90–8.20 (bs, 4H, C*H*_ar_) ppm. ^13^C{^1^H} NMR (126 MHz, 203 K, THF-*d*_8_): δ = 24.4 (bs, *C*H), 48.1 (d, *J*_PC_ = 6.3 Hz, *C*H), 55.0 (d, *J*_PC_ = 158.0 Hz, S*C*S), 122.5 (q, *J*_FC_ = 321.9 Hz, *C*F_3_), 128.2 (s, *C*H_ar_), 130.4 (s, *C*H_ar_), 131.4 (s, *C*H _ar_), 138.3 (s, *C*_ipso_) ppm. ^31^P{^1^H} NMR (121 MHz, 298 K, C_6_D_6_): δ = 25.7 ppm. ^19^F{^1^H} NMR (282 MHz, 298 K, C_6_D_6_): δ = −76.4 ppm. HRMS (ESI+): *m*/*z* [M+H]^+^ calculated for C_26_H_38_F_3_N_2_O_3_PS_2_ = 579.2092 found = 579.2104.

Intermediate **8**: Variable temperature analysis was performed at −70 °C in THF-*d*_8_. Data attributed to intermediate **8** were extracted from the crude analysis. ^1^H NMR (400 MHz, 203 K, THF-*d*_8_): δ = 0.70–0.90 (bs, 8H, C*H*_3_), 1.10–1.40 (bs, 16H, C*H*_3_), 3.50–4.00 (bs, 4H, C*H*), 7.20–8.00 (m, 20H, C*H*_ar_) ppm. ^13^C{^1^H} NMR (126 MHz, 203 K, THF-*d*_8_): δ = 24.4 (bs, *C*H_3_), 24.7 (bs, *C*H_3_), 34.5 (d, *J*_PC_ = 96.8 Hz, S*C*S), 47.1 (d, *J*_PC_ = 17.5 Hz *C*H), 49.6 (bs, *C*H), 122.4 (q, *J*_FC_ = 322.6 Hz, *C*F_3_), 128.7 (s, *C*H_ar_), 131.3 (s, *C*H_ar_), 133.7 (s, *C*H_ar_), 139.1 (s, *C*_ipso_) ppm. ^31^P{^1^H} NMR (121 MHz, 203 K, THF-*d*_8_): δ = 46.1 ppm.

### 3.3. X-ray Data

The data of the structures for **3**, **5**, **6-I**, **6-OTf**, and **7** were collected at low temperature (193 K) on a Bruker-AXS APEX II CCD Quazar diffractometer (**7**) equipped with a 30 W air-cooled microfocus source and on a Brucker-AXS D8-Venture diffractometer (**3**, **5**, **6-I**, and **6-OTf**) equipped with a Photon III-C14 detector with MoKα radiation (wavelength = 0.71073 Å) by using phi- and omega-scans. The data were integrated with SAINT, and an empirical absorption correction with SADABS was applied [48]. The structures were solved using an intrinsic phasing method (ShelXT) [49] and refined using the least-squares method on *F*^2^ (ShelXL-2014) [50]. All non-H atoms were treated anisotropically. All H atoms attached to C atoms were fixed geometrically, and treated as riding on their parent atoms with C-H = 0.95 Å (aromatic), 0.98 Å (CH_3_), 0.99 Å (CH_2_), or 1.0 Å (CH) with U_iso_ (H) = 1.2U_eq_ (CH, CH_2_) or U_iso_ (H) = 1.5U_eq_ (CH_3_). The structures **3** and **6-I** were found to be disordered. Several restraints (SAME, SADI, SIMU, DELU) were applied to refine these disorders, and to avoid the collapse of the structure during the least-squares refinement by the large anisotropic displacement parameters.

Supplementary crystallographic data for CCDC-2250100 (**3**), CCDC-2250101 (**5**), CCDC-2250102 (**6-I**), CCDC-2250103 (**6-OTf**), CCDC-2250104 (**7**) can be obtained free of charge from The Cambridge Crystallographic Data Centre via https://www.ccdc.cam.ac.uk/structures/.

## 4. Conclusions

In summary, S,S-bis-ylide **2** readily reacted with MeI to give the C-methylated salt **3**. Moreover, **2** also reacted with CO_2_ to give betaine **4**, which was characterized in solution by using NMR spectroscopy at low temperatures. CO_2_ adduct **4** was still reactive and underwent O-silylation and O-alkylation reactions in the presence of HMDS and MeI, respectively, to afford the corresponding salts **5** and **6**. Finally, the high leaving group ability of sulfide ligand in **2** was demonstrated by an original reaction with phosphenium ions, yielding a stabilized ylide **7**, probably through the transient formation of a strongly electrophilic phosphino(sulfonio)carbene **9**. Efforts are currently underway to extend the potential of this type of ylide.

## Data Availability

Not applicable.

## Supplementary Materials

The following supporting information can be downloaded at: https://www.mdpi.com/article/10.3390/molecules28083295/s1, NMR spectra and crystallographic data.

## Sample Availability

Samples of the compounds are not available from the authors.
