# Supplementary material for: New Insight into the Reactivity of S,S-Bis-ylide
Source: Molecules. 2023 Apr 7;28(8):3295. doi: 10.3390/molecules28083295 (PMC10142965; doi:10.3390/molecules28083295)
Supplement: Supplementary file 1 [file molecules-28-03295-s001.zip › molecules-2342373-supplementary.pdf]

Supporting information for

New Insight into the Reactivity of S,S-Bis-ylide

Ugo Authesserre [1†], V. S. V. S. N. Swamy [1†], Nathalie Saffon-Merceron [2], Antoine Baceiredo [1], Tsuyoshi Kato [1,*], and Eddy Maerten [1,*]

[1] Université de Toulouse, UPS, and CNRS, LHFA UMR 5069, 118 Route de Narbonne, 31062 Toulouse, France
[2] Université de Toulouse, UPS, and CNRS, ICT UAR2599 118 Route de Narbonne, 31062 Toulouse, France
* Correspondence: tsuyoshi.kato@univ-tlse3.fr (T.K.); eddy.maerten@univ-tlse3.fr (E.M.)
† These authors contributed equally to this work

	Page
**Table of contents**	**S2**
**Selected spectroscopic data**	**S3**
**X-ray analysis**	**S8**

Selected spectroscopic data

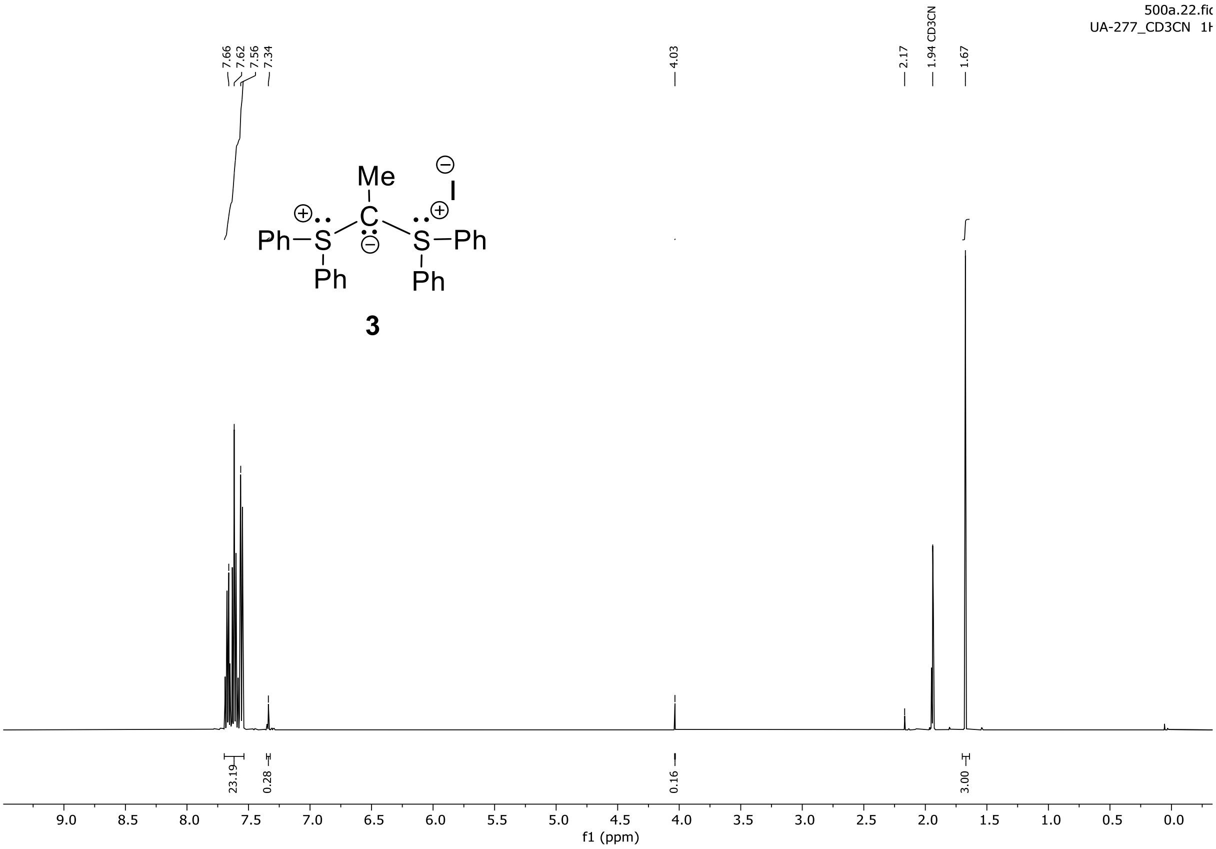

Figure S1. ^{1}H NMR (500 MHz, CD_3CN)

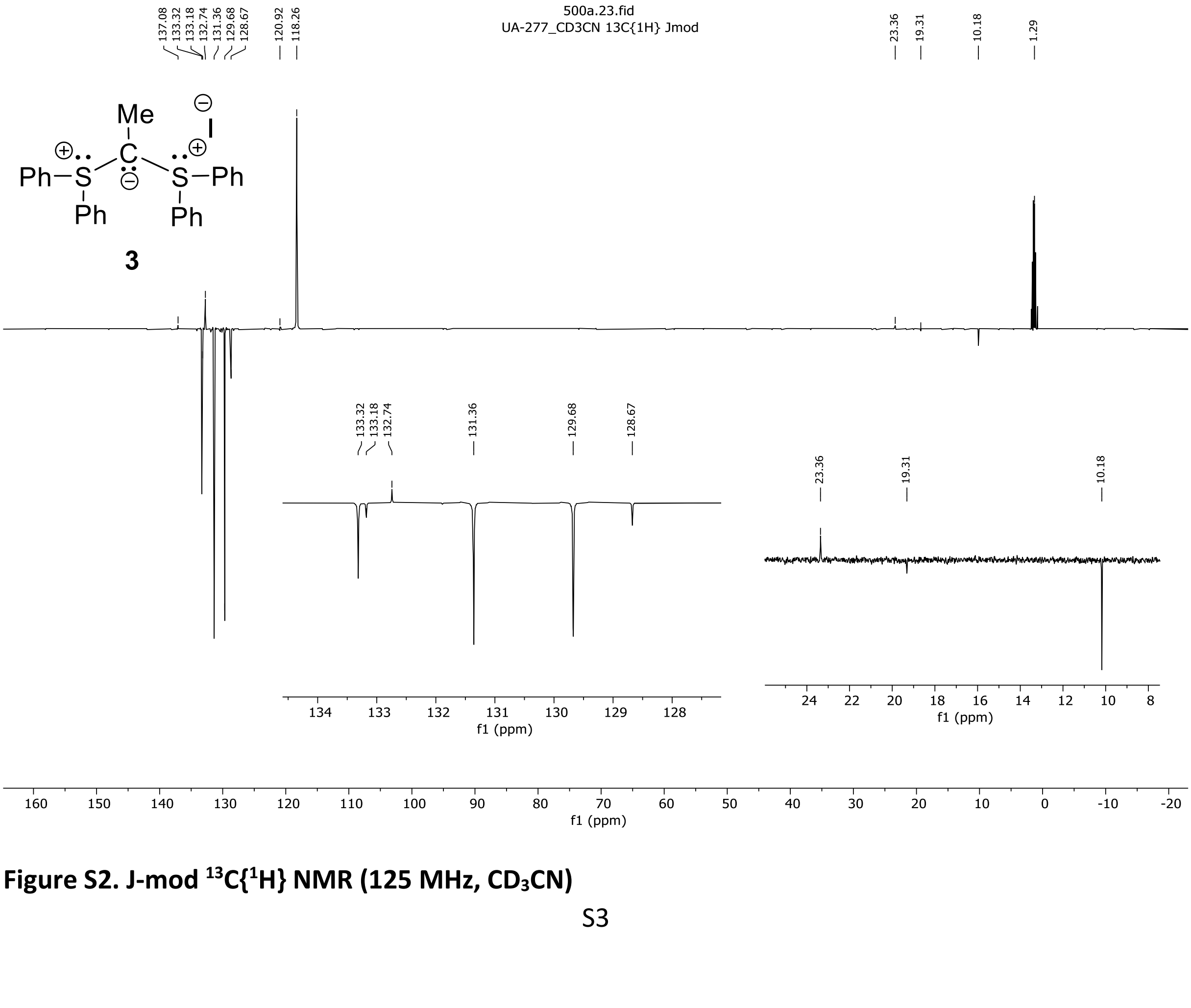

Figure S2. J-mod $^{13}C\{^{1}H\}$ NMR (125 MHz, CD_3CN)

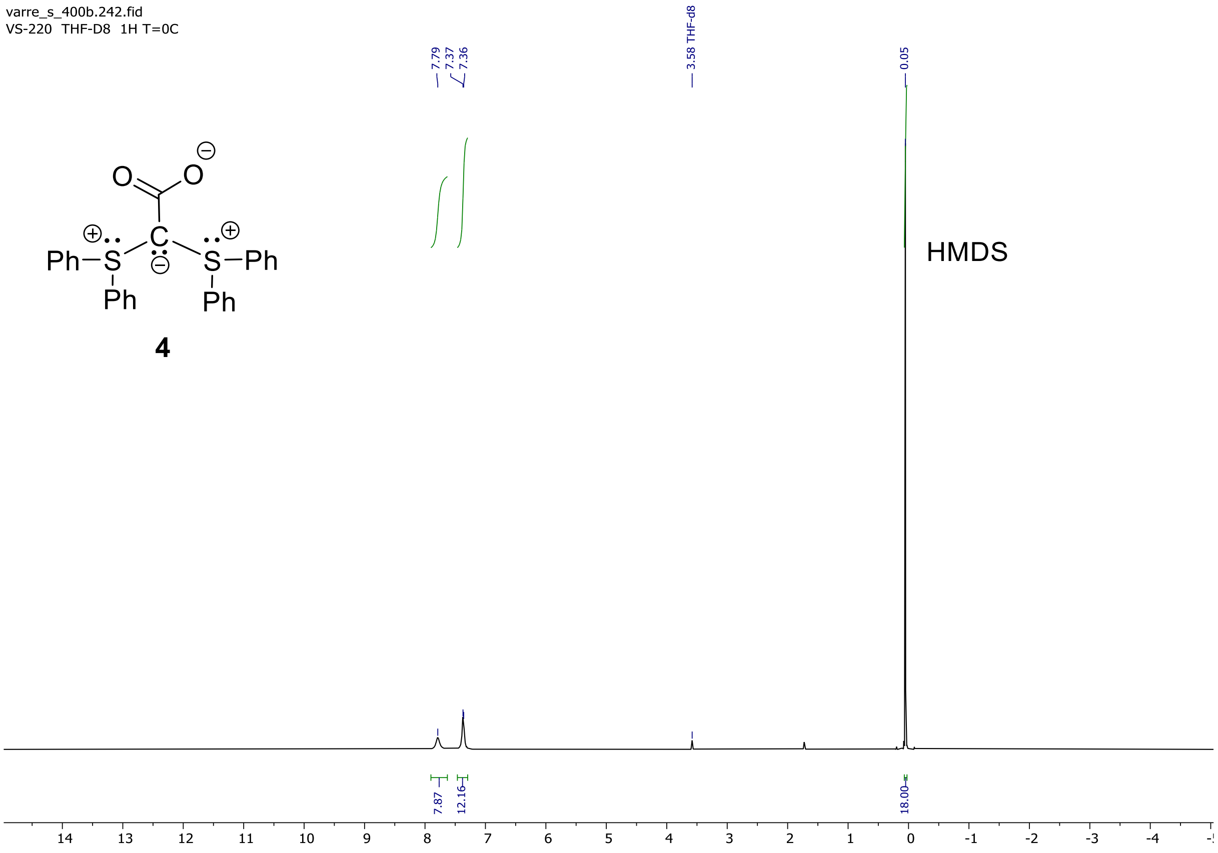

Figure S3. **^{1}H NMR (400 MHz, THF-d_8, 273 K)**

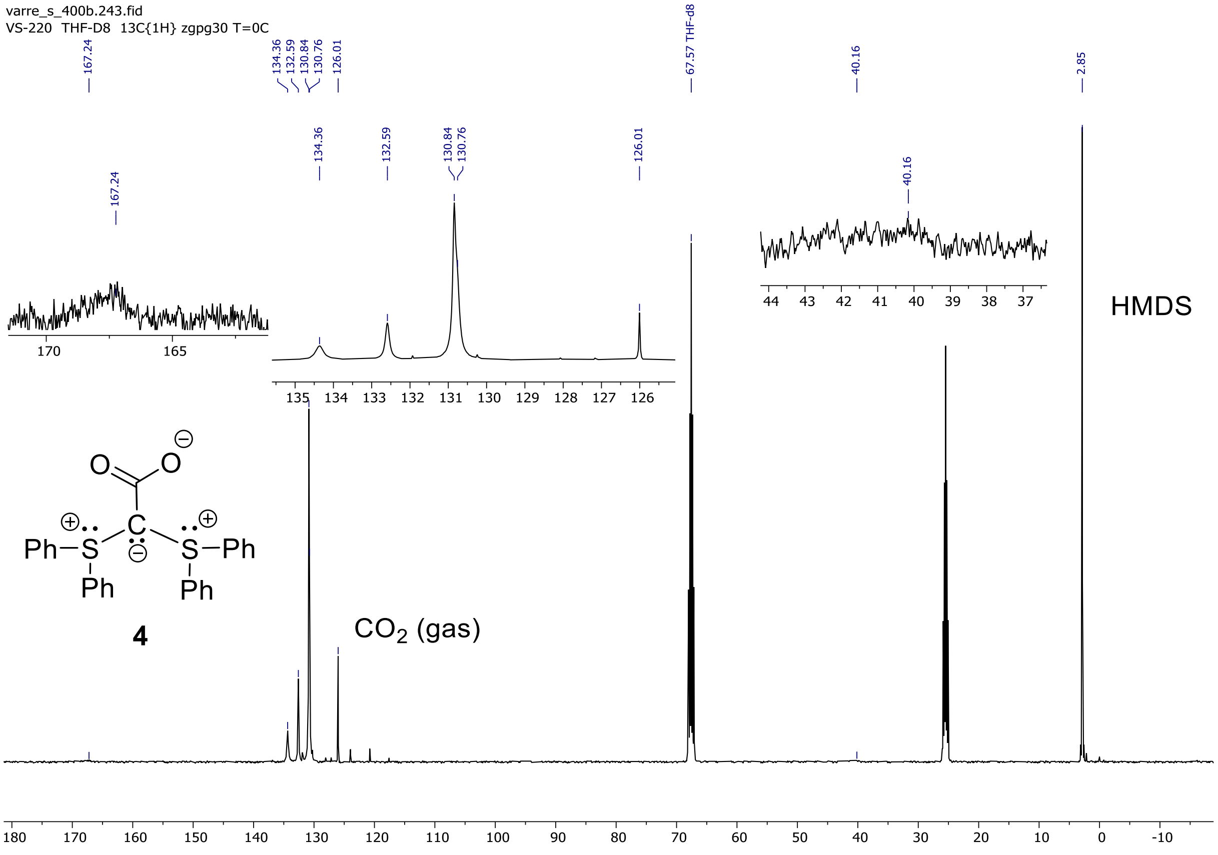

Figure S4. **^{13}C{1H} NMR (100 MHz, THF-d_8, 273 K)**

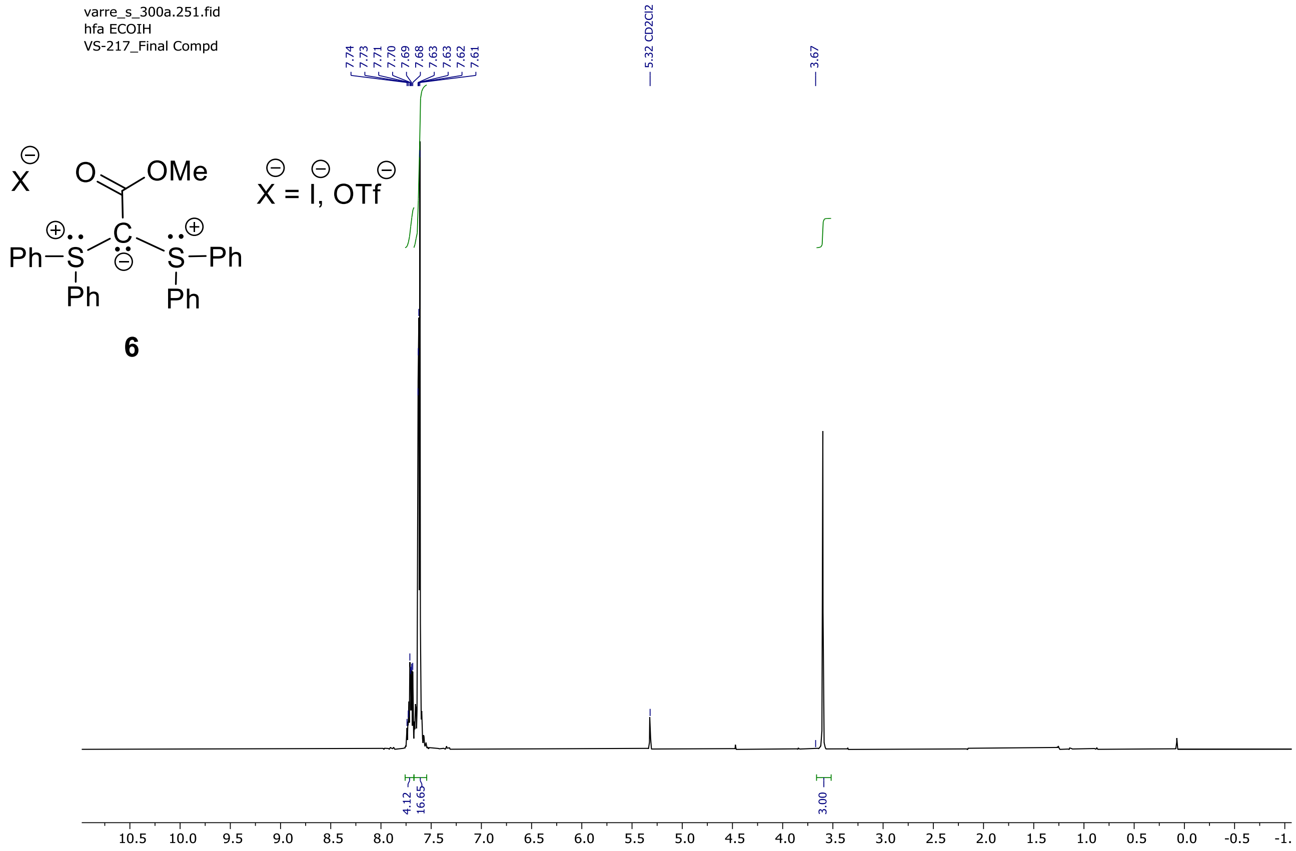

Figure S5. ^{1}H **NMR (300 MHz,** CD_2Cl_2**)**

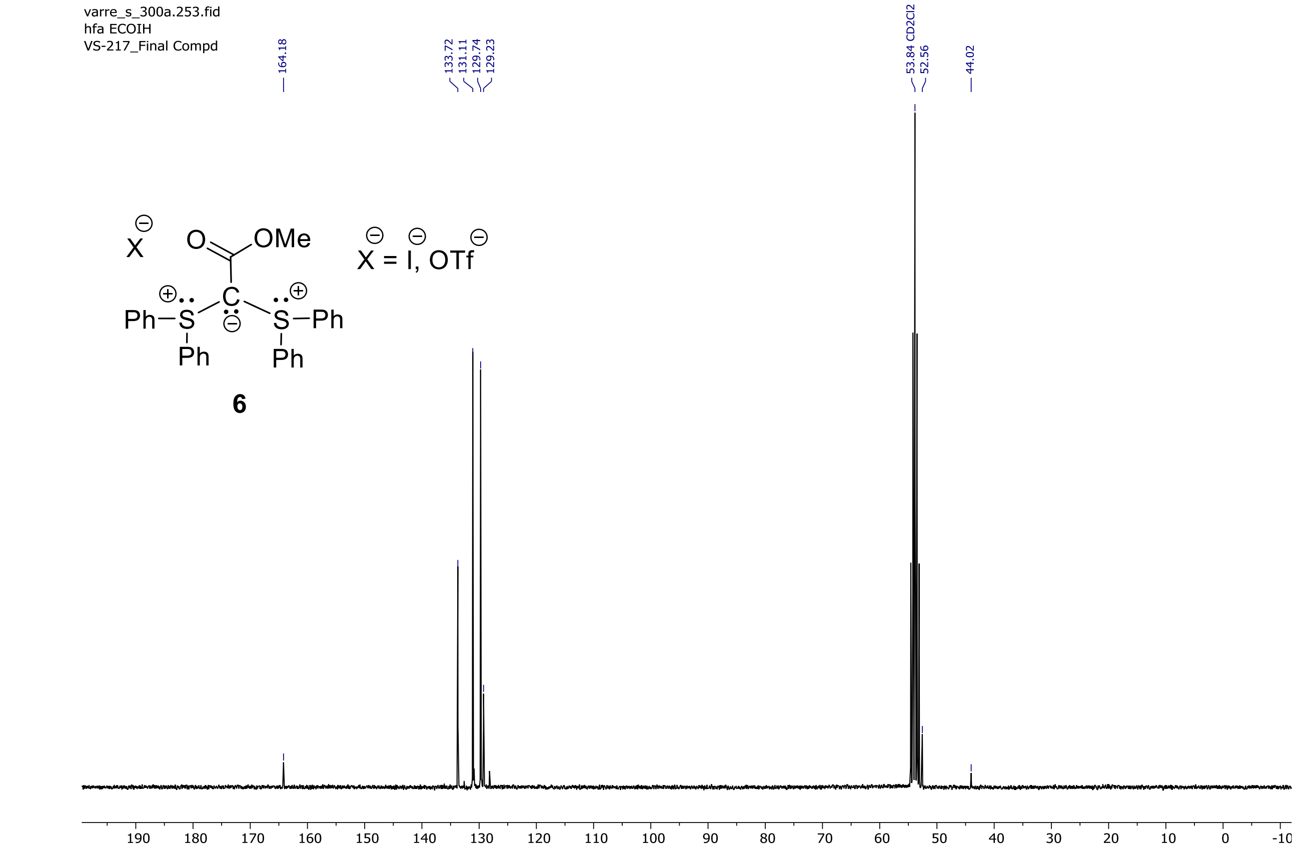

Figure S6. $^{13}C\{^{1}H\}$ **NMR (75 MHz,** CD_2Cl_2**)**

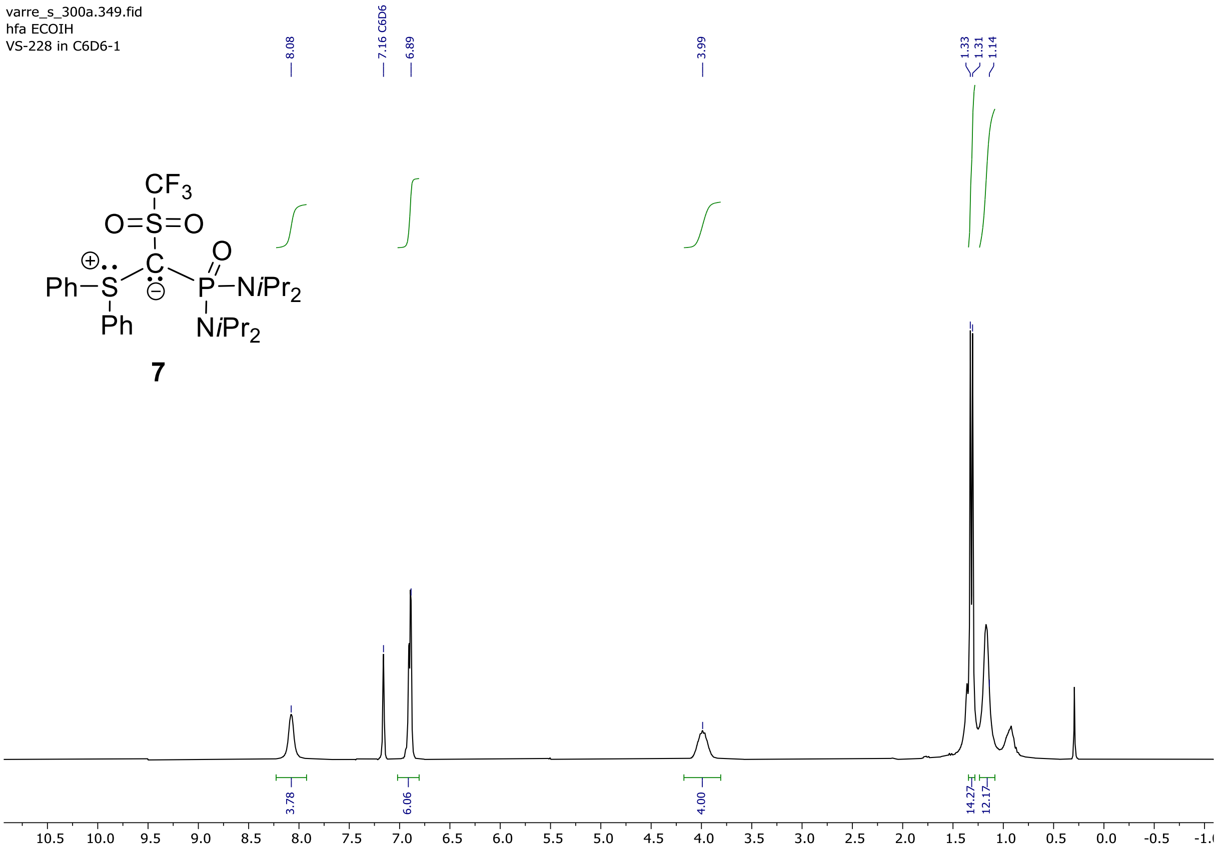

Figure S7. ^{1}H NMR (300 MHz, C_6D_6)

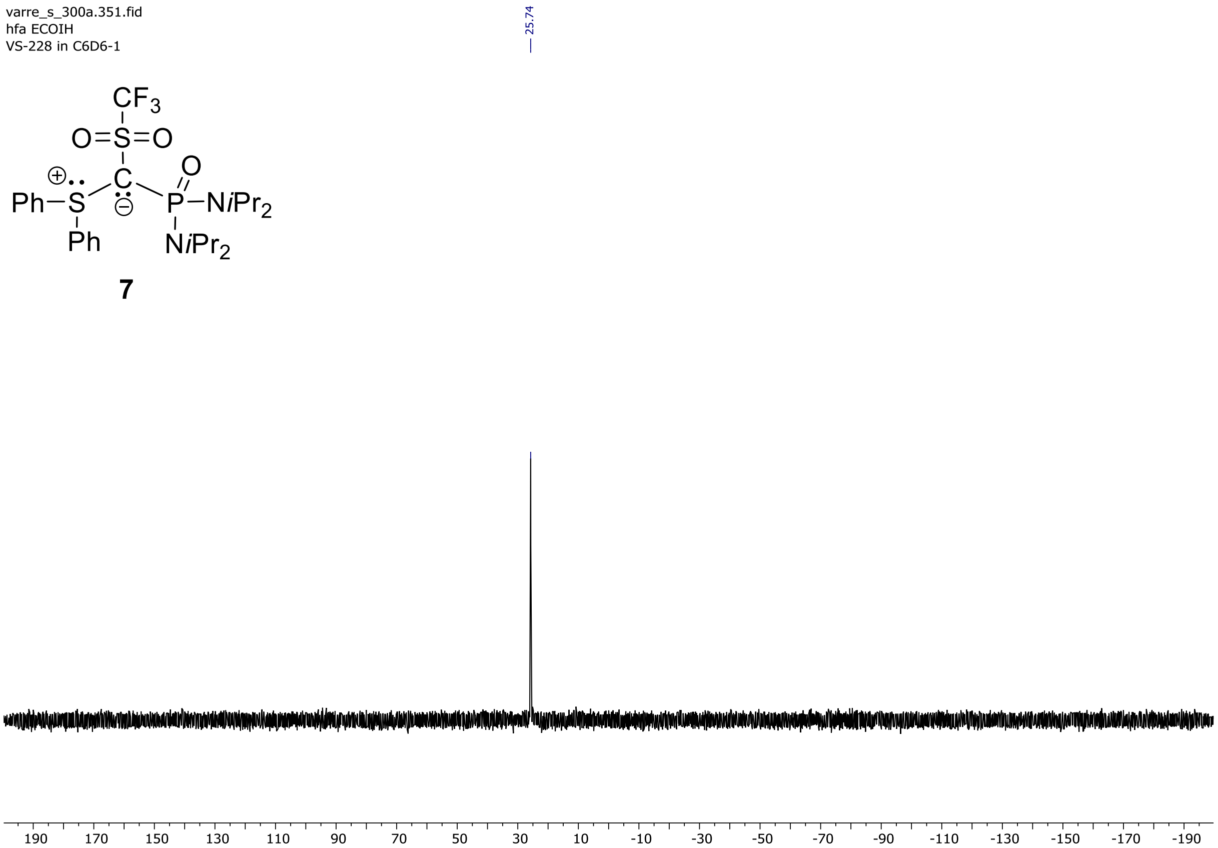

Figure S8. $^{31}P\{^{1}H\}$ NMR (121 MHz, C_6D_6)

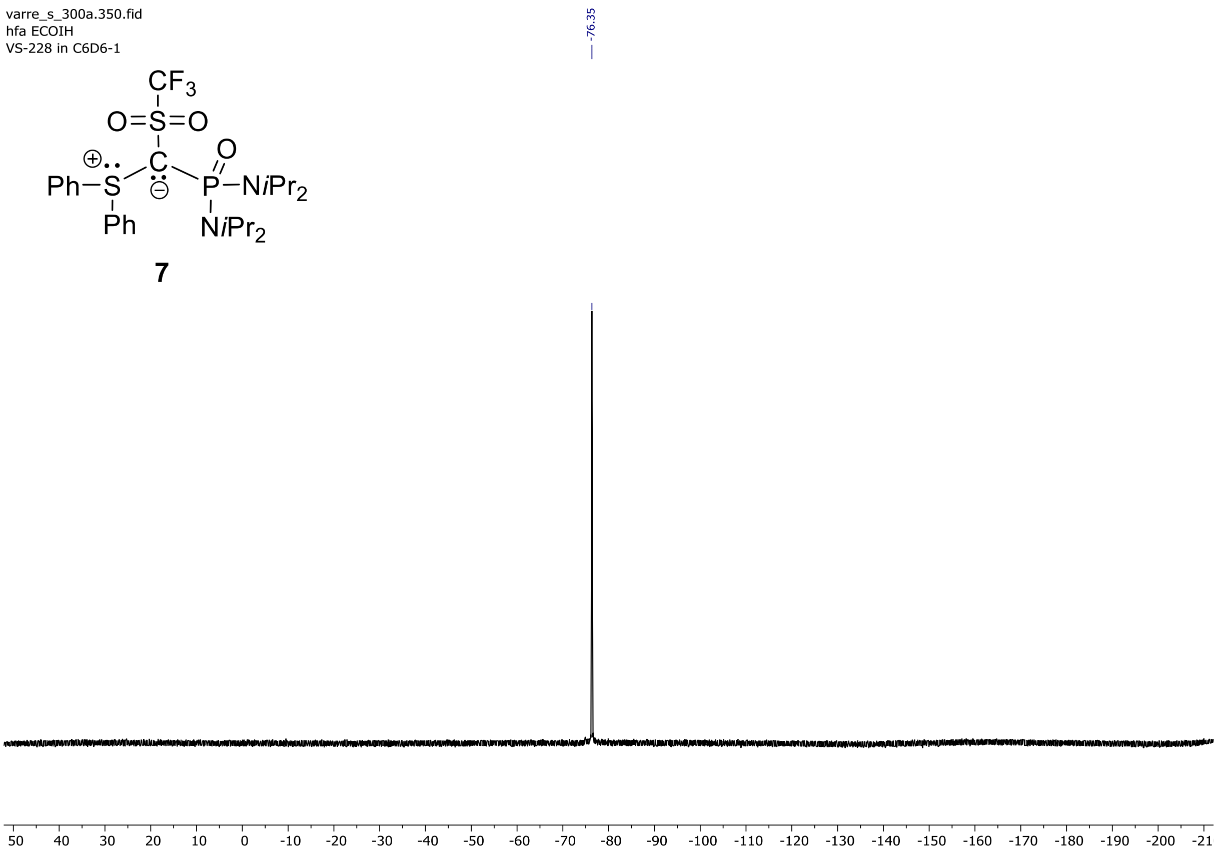

Figure S9. $^{19}F\{^1H\}$ **NMR (282 MHz,** C_6D_6**)**

X-ray analysis

The data of the structures **3**, **5, 6-I, 6-OTf** and **7** were collected at 193 K on a Bruker-AXS APEX II CCD Quazar diffractometer (**7**) equipped with a 30 W air-cooled microfocus source and on a Brucker-AXS D8-Venture diffractometer (**3**, **5, 6-I** and **6-OTf**) equipped with a Photon III-C14 detector with MoKα radiation (wavelength = 0.71073 Å) by using phi- and omega-scans. The data were integrated with SAINT, and an empirical absorption correction with SADABS was applied [S1]. The structures were solved using an intrinsic phasing method (ShelXT) [S2] and refined using the least–squares method on F^2 (ShelXL-2014) [S3]. All non-H atoms were treated anisotropically. All H atoms attached to C atoms were fixed geometrically and treated as riding on their parent atoms with C-H = 0.95 Å (aromatic), 0.98 Å (CH_3), 0.99 Å (CH_2) or 1.0 Å (CH) with $U_{iso}(H) = 1.2U_{eq}(CH, CH_2)$ or $U_{iso}(H) = 1.5U_{eq}(CH_3)$. The structures **3** and **6-I** were found to be disordered. Several restraints (SAME, SADI, SIMU, DELU) were applied to refine these disorders and to avoid the collapse of the structure during the least-squares refinement by the large anisotropic displacement parameters.

Supplementary crystallographic data for CCDC-2250100 (**3**), CCDC-2250101 (**5**), CCDC-2250102 (**6-I**), CCDC-2250103 (**6-OTf**) and CCDC-2250104 (**7**) can be obtained free of charge from The Cambridge Crystallographic Data Centre via https://www.ccdc.cam.ac.uk/structures/.

The details of data collection and crystal structures refinement are summarized in Table S1 and S2.

Table S1. Crystallographic data for the compounds **3**, **5** and **6-I**

**Compound**	**3**	**5**	**6-I**
Chemical formula	$C_{26}H_{23}IS_2$	$C_{30}H_{29}F_3O_5S_3Si$	$C_{27}H_{23}IO_2S_2$
M_r	526.46	650.80	570.47
Crystal system	Monoclinic	Monoclinic	Monoclinic
Space group	C2/c	*P2₁*/c	C2/c
a [Å]	19.4524(6)	10.5839(5)	21.7134(17)
b [Å]	10.1958(4)	14.1490(7)	15.8295(13)
c [Å]	14.0908(5)	20.5519(8)	17.2334(13)
α [°]	90	90	90
β [°]	123.8931(10)	92.8708(18)	123.179(2)
γ [°]	90	90	90
V [$Å^3$]	2319.80(14)	3073.8(2)	4957.6(7)
Z	4	4	8
ρ [g cm^{-3}]	1.507	1.406	1.529
$\mu(Mo_{K\alpha})$ [mm^{-1}]	1.570	0.337	1.482
Reflections collected	41092	169695	123535
Independent reflections	2859 R(int)=0.0339	7647 R(int)=0.0567	6152 R(int)=0.0719
Data/ restraints/ parameters	2859/293/210	7647/0/382	6152/226/345
Crystal size [mm^3]	0.200x0.200x0.150	0.220×0.200×0.040	0.200×0.150×0.130
GOOF on F^2	1.241	1.039	1.051
R (I > 2σ(I))	0.0396	0.0342	0.0341
wR2 (all data)	0.0945	0.0922	0.0831
Largest difference peak and hole, [e $Å^{-3}$]	0.794 and -0.629	0.399 and -0.308	1.315 and -0.831
CCDC number	2250100	2250101	2250102

Table S2. Crystallographic data for the compounds **6-OTf** and **7**

**Compound**	**6-OTf**	**7**
Chemical formula	$C_{28}H_{23}F_3O_5S_3$	$C_{26}H_{38}F_3N_2O_3PS_2$
M_r	592.64	578.67
Crystal system	Triclinic	Monoclinic
Space group	$P\bar{1}$	$P2_1/n$
a [Å]	8.4599(5)	9.7299(11)
b [Å]	10.1945(8)	25.933(3)
c [Å]	15.6864(11)	11.4278(14)
α [°]	87.221(3)	90
β [°]	80.592(3)	93.745(3)
γ [°]	81.145(3)	90
V [Å^3]	1318.38(16)	2877.4(6)
Z	2	4
ρ [g cm^{-3}]	1.493	1.336
$\mu(Mo_{K\alpha})$ [mm^{-1}]	0.341	0.290
Reflections collected	47401	94369
Independent reflections	6600 R(int)=0.0673	7249 R(int)=0.1047
Data/ restraints/ parameters	6600/0/353	7249/0/342
Crystal size [mm^3]	0.200x0.120x0.050	0.200×0.200×0.050
GOOF on F^2	1.051	1.031
R (I > 2σ(I))	0.0421	0.0400
wR2 (all data)	0.1028	0.0914
Largest difference peak and hole, [e Å^{-3}]	0.640 and -0.296	0.327 and -0.350
CCDC number	2250103	2250104

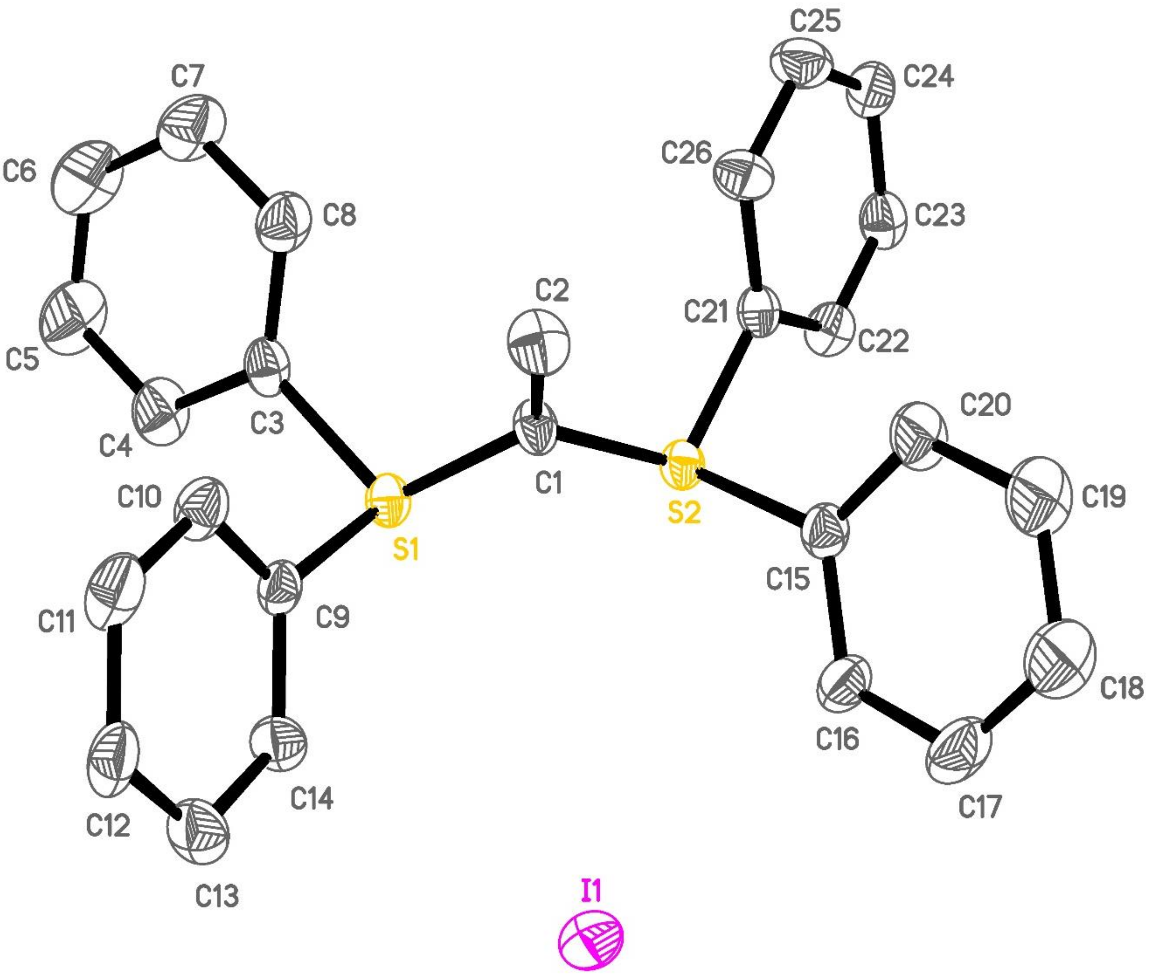

Figure S10. Molecular view of **3**. Thermal ellipsoids set at 30 % probability. H and disordered atoms are omitted for clarity. Selected bond lengths (Å) and angles (°): S1-C1 1.682(9), C1-S2 1.687(9), C1-C2 1.525(12), S1-C3 1.794(4), S1-C9 1.759(3), S2-C21 1.772(4), S2-C15 1.776(3). S1-C1-S2 111.6(6), S2-C1-C2 123.8(6), C2-C1-S1 124.3(6), C9-S1-C1 107.4(3), C1-S1-C3 111.6(4), C3-S1-C9 100.2(2), C1-S2-C15 107.6(3), C15-S2-C21 101.5(2), C21-S2-C1 111.9(4).

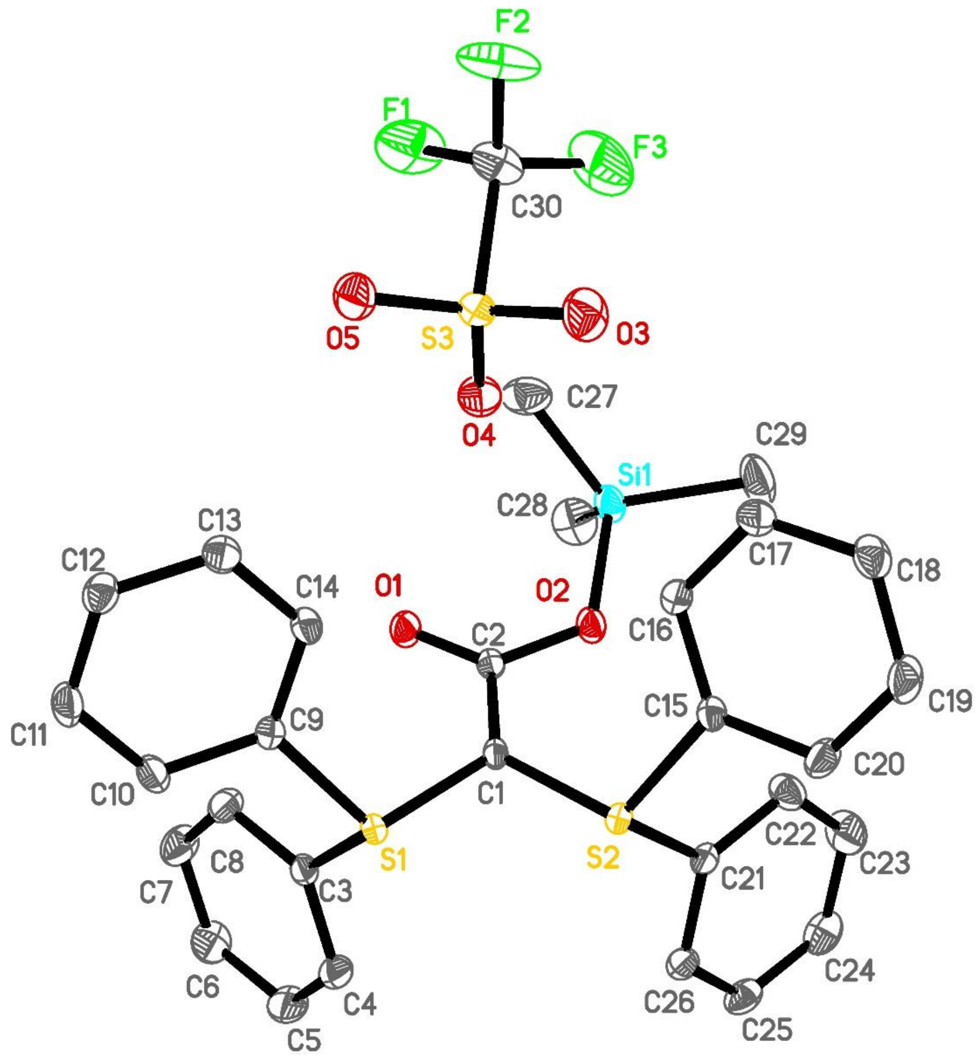

Figure S11. Molecular view of **5**. Thermal ellipsoids set at 30 % probability. H atoms are omitted for clarity. Selected bond lengths (Å) and angles (°): S1-C1 1.717(2), C1-S2 1.717(1), C1-C2 1.449(2), C2-O1 1.218(2), C2-O2 1.344(2), O2-Si1 1.705(1), Si1-C27 1.846(2), Si1-C28 1.847(2), Si1-C29 1.846(2), S1-C3 1.790(2), S1-C9 1.792(2), S2-C21 1.790(2), S2-C15 1.790(1). S1-C1-S2 110.62(8), S2-C1-C2 126.62(10), C2-C1-S1 122.00(10), C1-C2-O1 123.83(13), O1-C2-O2 123.36(13), O2-C2-C1 112.81(11).

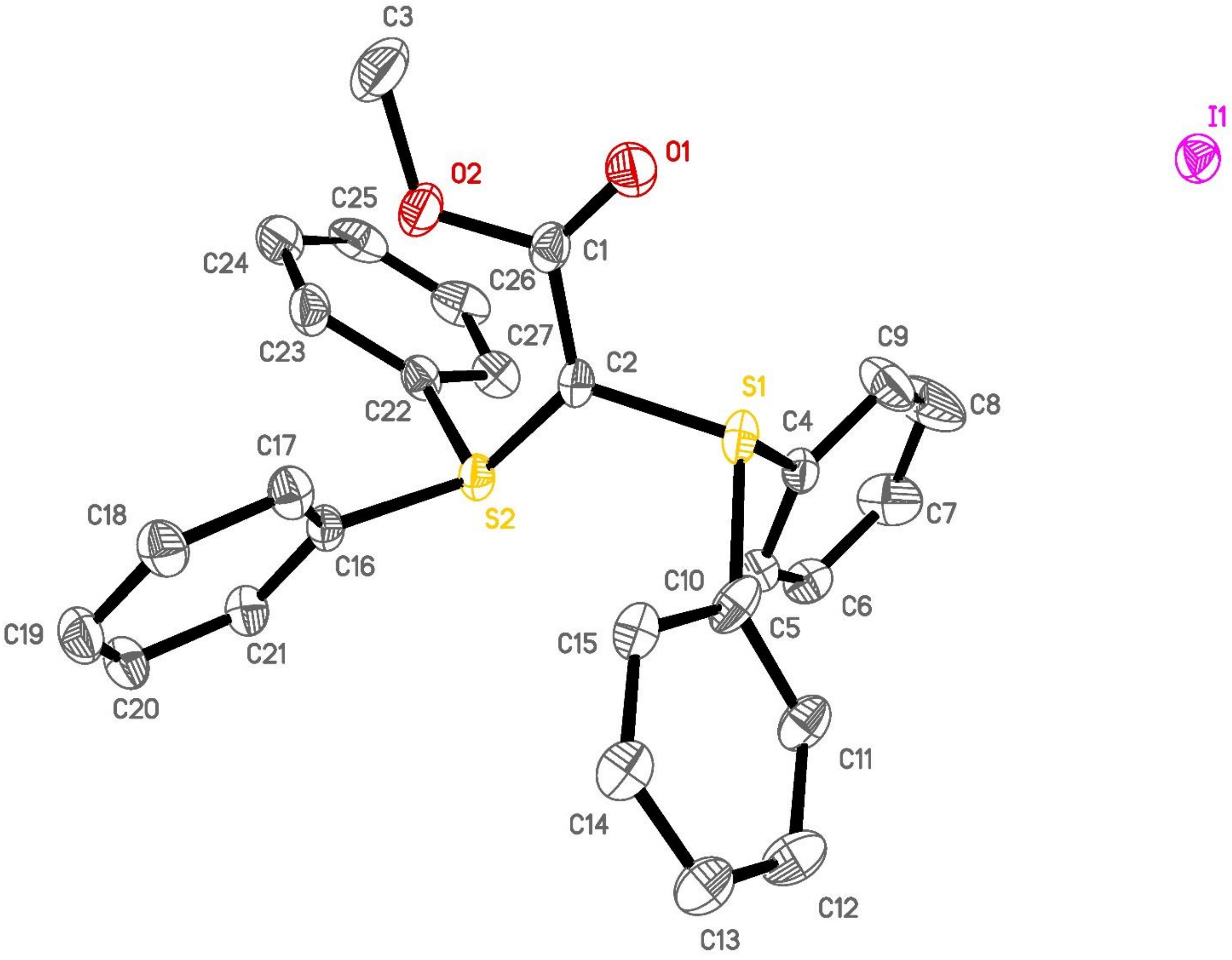

Figure S12. Molecular view of **6-I**. Thermal ellipsoids set at 30 % probability. H and disordered atoms are omitted for clarity. Selected bond lengths (Å) and angles (°): S1-C2 1.720(2), C2-S2 1.713(3), C1-C2 1.437(4), C1-O1 1.214(3), C1-O2 1.351(3), O2-C3 1.438(4), S1-C4 1.783(3), S1-C10 1.803(5), S2-C22 1.793(3), S2-C16 1.794(3). S1-C2-S2 117.5(1), S2-C2-C1 127.6(2), C1-C2-S1 114.1(2), C2-C1-O2 110.8(2), O2-C1-O1 122.6(3), O1-C1-C2 126.6(2).

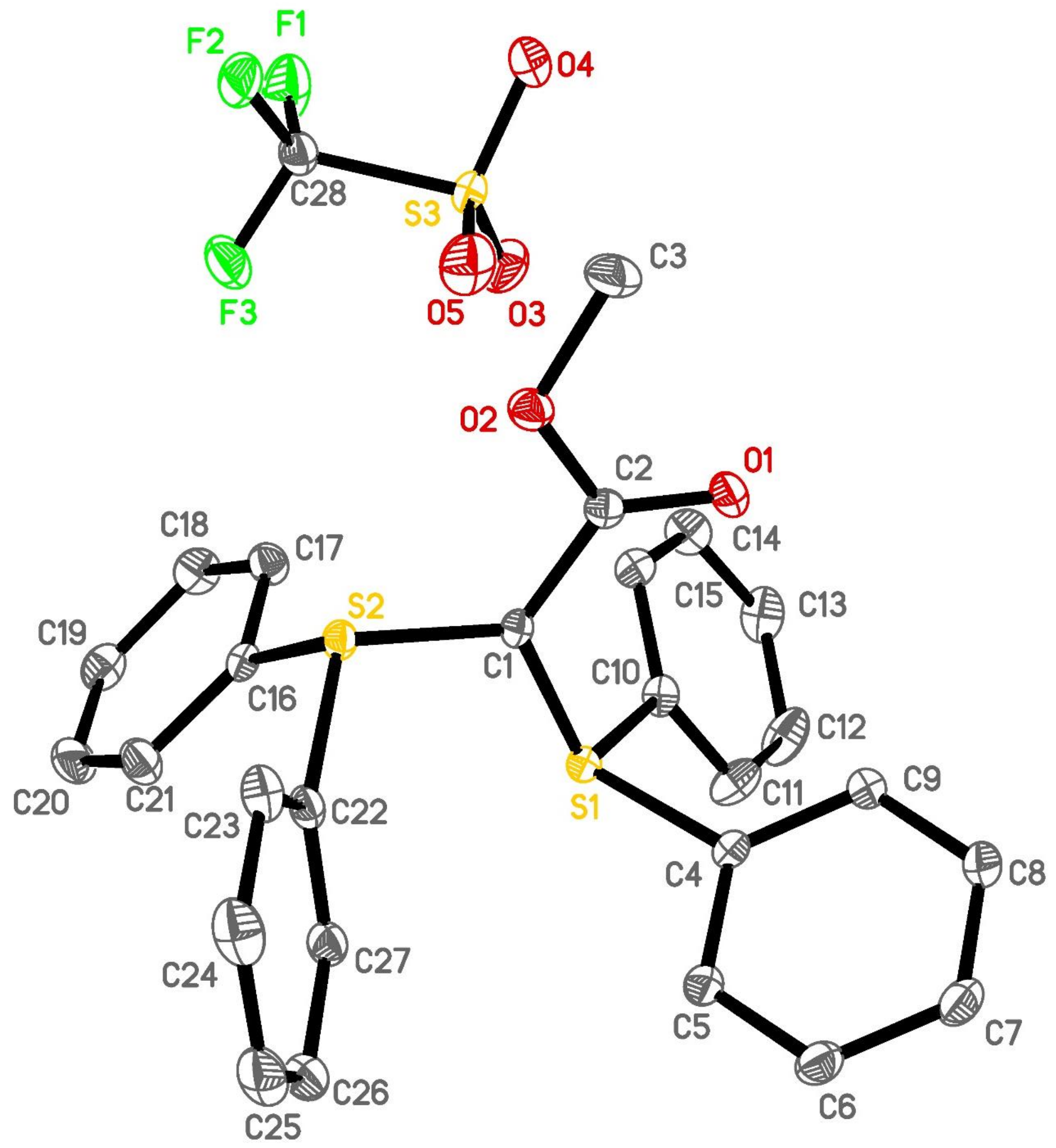

Figure S13. Molecular view of **6-OTf**. Thermal ellipsoids set at 30 % probability. H atoms are omitted for clarity. Selected bond lengths (Å) and angles (°): S1-C1 1.708(2), C1-S2 1.719(2), C1-C2 1.438(3), C2-O1 1.213(2), C2-O2 1.351(2), O2-C3 1.439(3), S1-C4 1.792(2), S1-C10 1.795(2), S2-C22 1.785(2), S2-C16 1.793(2). S1-C1-S2 118.17(11), S1-C1-C2 122.30(14), C2-C1-S2 119.27(14), C1-C2-O2 112.64(16), O2-C2-O1 123.38(18), O1-C2-C1 123.97(18).

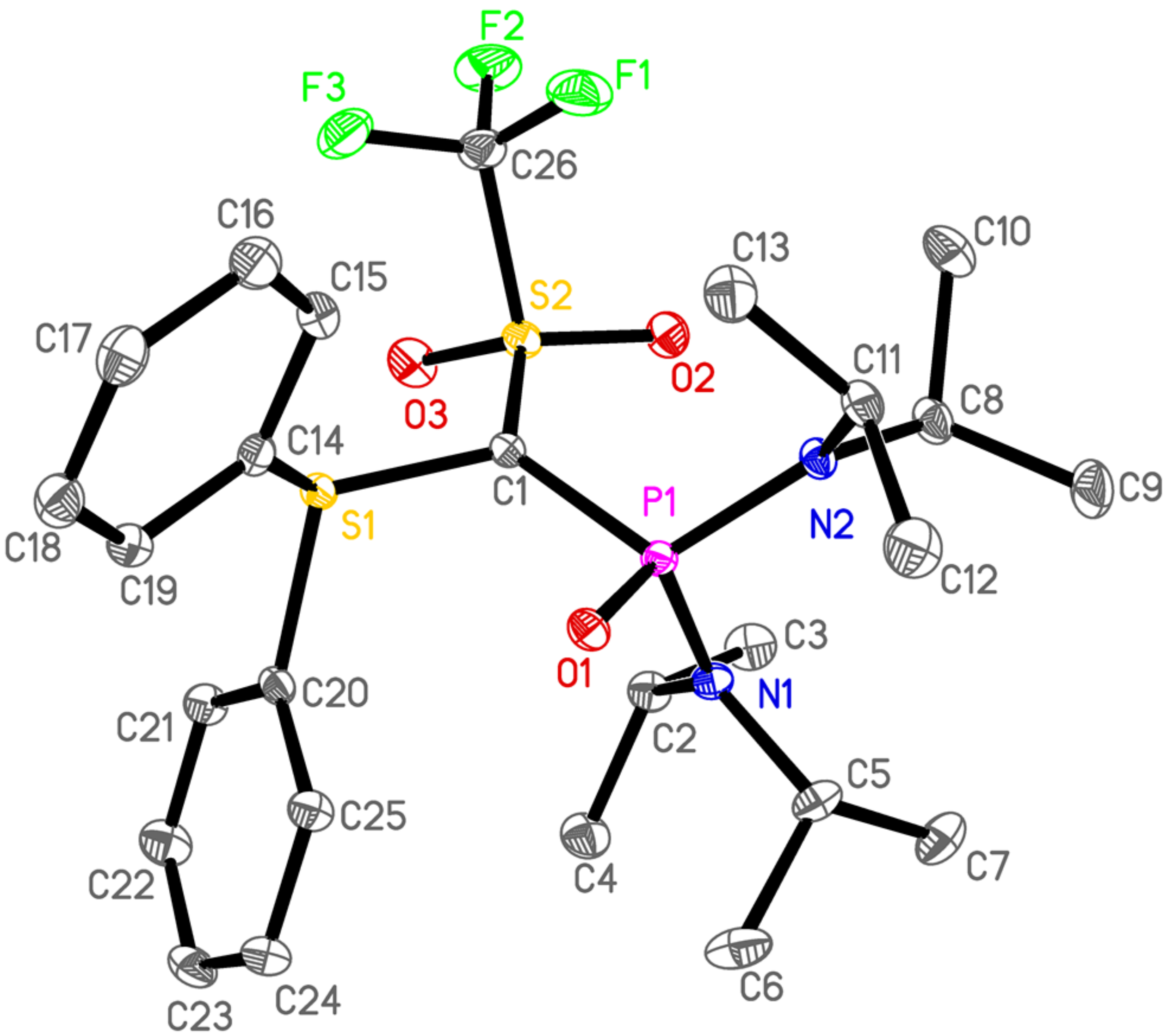

Figure S14. Molecular view of **7**. Thermal ellipsoids set at 30 % probability. H atoms are omitted for clarity. Selected bond lengths (Å) and angles (°): S1-C1 1.724(2), C1-S2 1.694(2), C1-P1 1.807(2), S1-C14 1.803(2), S1-C20 1.788(2), P1-O1 1.487(1), P1-N1 1.669(2), P1-N2 1.656(2), S2-O2 1.434(2), S2-O3 1.437(1), S2-C26 1.841(2), C26-F1 1.327(2), C26-F2 1.336(2), C26-F3 1.333(2). S1-C1-P1 120.80(10), S1-C1-S2 109.97(10), P1-C1-S2 127.57(11).

S1 SADABS, Program for data correction, Bruker−AXS.
S2 Sheldrick, G. M.; *SHELXT* ; Integrated space-group and crystal-structure determination. *Acta Crystallogr.* 386 *Sect. A*, **2015**, *71*, 3-8.
S3 Sheldrick; G. M.; Crystal structure refinement with *SHELXL*. *Acta Crystallogr. Sect. C*, **2015**, *71*, 3-8.
